# Investigation of changes in structure and thermodynamic of spruce budworm antifreeze protein under subfreezing temperature

**DOI:** 10.1038/srep40032

**Published:** 2017-01-20

**Authors:** Hung Nguyen, Ly Le

**Affiliations:** 1 Open Lab, Institute for Computational Sciences and Technology at Ho Chi Minh City, Vietnam; 2 School of Biotechnology, International University, Vietnam National University at Ho Chi Minh, Vietnam

## Abstract

The aim of this theoretical work is to investigate of the changes in structure and thermodynamics of spruce budworm antifreeze protein (sbAFP) at low temperatures by using molecular dynamics simulation. The aqueous solution will form ice crystal network under the vaguely hexagonal shape at low temperature and fully represented the characteristics of hydrophobic interaction. Like ice crystal network, the cyclohexane region (including cyclohexane molecules) have enough of the characteristics of hydrophobic interaction. Therefore, in this research the cyclohexane region will be used as a representation of ice crystal network to investigate the interactions of sbAFP and ice crystal network at low temperature. The activity of sbAFP in subfreezing environment, therefore, can be clearly observed via the changes of the hydrophobic (cyclohexane region) and hydrophilic (water region) interactions. The obtained results from total energies, hydrogen bond lifetime correlation C(t), radial distribution function, mean square deviation and snapshots of sbAFP complexes indicated that sbAFP has some special changes in structure and interaction with water and cyclohexane regions at 278 K, as being transition temperature point of water molecules in sbAFP complex at low temperatures, which is more structured and support the experimental observation that the sbAFP complex becomes more rigid as the temperature is lowered.

Organisms living in subfreezing regions have acquired the ability to adapt their living conditions by producing antifreeze proteins (AFPs) which can inhibit ice crystal growth process. AFPs are composed of only protein or in combination with glycan via covalent bonds, also called antifreeze glycoprotein (AFGP)^1^,^2^,^3^. AFPs protect their host by limiting the growth of ice crystal inside and outside body fluids^4^,^5^. AFPs and AFGPs have been found in various organism including fish^6^,^7^,^8^, plants^9^,^10^,^11^, insects^12^,^13^,^14^,^15^, fungi^16^, and bacteria^3^,^17^,^18^. The antifreeze mechanism of AFPs is determined through microscopic analysis which is showed that AFPs bind to ice surface and prevent ice crystallization process^19^,^20^,^21^. The difference between AFPs’ melting point and freezing point is known as thermal hysteresis (TH), which is measured using a nanoliter osmometer and is also used to quantify the activity of AFPs and other IBPs^22^. Additionally, Meister *et al*. also found that the presence of ice-like water layers at the ice-binding site (IBS) of AFPs in aqueous solution at temperatures above the freezing point: Decreasing the temperature to the biological working temperature of AFPs (0 °C to −2 °C) led to increase in the amount of ice-like water, while a single point mutation in the IBS of AFPs was observed to completely disrupt the ice-like character and to eliminate their anti-freezing activities. This was not AFPs itself but ordered ice-like water layer that are responsible for the recognition and binding to ice crystal^23^. In another research, Duboué-Dijon and Laage pointed out that the average perturbation induced by hyperactive antifreeze protein and ubiquitin on the reorientation dynamics of water remains moderate and changes weakly with temperature. The ice-binding surface of the antifreeze protein induces a short ranged enhancement of water structure and a greater slowdown of water reorientation dynamics than the non-ice-binding surfaces whose effect is similar to that of ubiquitin; and the hydration shell of the ice-binding surface remains less tetrahedral than the bulk and is not ice-like. And the hydrogen bonds between water and the ice-binding threonine residues are particularly strong due to a steric confinement effect, thereby contributing to the strong binding of the antifreeze protein on ice crystals^24^. Moreover, previous studies also showed that ice growth is kinetically inhibited by AFPs covering the water accessible surfaces of ice^25^,^26^,^27^. AFPs are particularly sensitive to cooling condition as their molecular adaptation to ice crystal is induced through the gradual formation of ice cascade surrounding AFP which can also be interpreted as AFPs can be inactivated if subfreezing temperature suddenly drops^28^,^29^,^30^,^31^.

The IBS of AFPs has been found to be relatively hydrophobic and also contained many potential hydrogen bond donors/acceptors^32^. The idea of hydrogen bonds and the hydrophobic effects contribute to ice-binding has been under investigation in recent decades^33^. Hydrogen bonds were originally proposed to be the major interaction between AFPs and ice crystal^34^. However, this hypothesis failed to provide a rational explanation on how an AFP would preferably bind ice crystal. On the other hand, recent studies proposed that the hydrophobic effect could have played an important function in the ice inhibition process and provided with evidence that clathrate-like water on the hydrophobic IBS was found to be released into the solvent upon ice-binding, resulting in a gain in entropy^35^,^36^. In addition, molecular dynamics simulation studies indicated that the relatively hydrophobic IBS of AFPs is capable of reordering water molecules into an ice-like lattice and, instead of shedding bound water molecules upon ice-binding, the reordered water molecules might facilitate the AFP’s interaction with ice by matching certain ice geometrical planes^37^. The conformational rigidity of the hyperactive *Tenebrio molitor* antifreeze protein (*Tm*AFP) in aqueous medium and the structural arrangements of water molecules hydrating its surface (simulated at 300 and 220 K) also found that irrespective of the temperature the IBS of the protein is relatively more rigid than its non-ice-binding surface is the presence of a set of regularly arranged internally bound water molecules is found to play a significant role in maintaining the flat rigid nature of the IBS. Importantly, their study also revealed that the strategically located hydroxyl oxygens of the threonine residues in the IBS influence the arrangements of five sets of ordered waters around it on two parallel planes that closely resemble the basal plane of ice. These water molecules can register well with the ice basal plane, thereby allowing the IBS to preferentially bind at the ice interface and inhibit its growth. This provides a possible reason behind the ice-binding activity of *Tm*AFP at the basal plane of ice^26^,^38^. Although intriguing as it seems, these simulations failed to describe the molecular mechanism explaining how an AFP acquires its ability to reorder water molecules in which binding specificity and affinity can assist in the irreversible absorption of AFP into forming ice lattices. These problems have been elucidated by the ice-binding mechanisms and the first crystal structure of Antarctic bacterium AFPs. The largest AFP experimental structures demonstrated that folds or Ca^2+^-bound parallel beta-helices with an extensive array of ice-like surfaces were directly anchored via hydrogen bonds to the polypeptide backbone and the adjacent side chains^32^,^34^,^37^,^38^,^39^,^40^,^41^,^42^,^43^.

Kuiper *et al*. previously studied that the protein structure-function mechanism for the spruce budworm *Choristoneura fumiferana* AFP, including stereo-specific binding, consequential melting, and freezing inhibition. The molecular details of AFP adsorption inhibition is uncertain but is proposed to involve the Gibbs-Thomson effect^44^. The protein binds indirectly to the prism ice surface through a linear array of ordered water molecules that are structurally distinct from the ice^45^. And mutation of the ice-binding surface disrupts water ordering and abolishes activity^46^. In another research, Steffen P. Graether *et al*. reported that the first structures of the highly active insect AFPs has been characterized. These proteins have a b-helix structure, which adds yet another fold to the AFP family. The 90 residue spruce budworm (*Choristoneura fumiferana*) AFP consists of a β-helix with 15 residues per coil. The structure contains two ranks of aligned threonine residues (known as the TXT motif), which were shown by mutagenesis experiments to be located in the ice-binding surface. In their previous NMR study of this AFP at 30 °C, the TXT surface was not optimally defined because of the broadening of NMR resonances potentially due to weak oligomerization. The structure of spruce budworm AFP was determined at 5 °C, where this broadening is reduced. In addition, the ^1^H-^15^N NMR dynamics of the protein were examined at 30 °C and 5 °C. The results show that the spruce budworm AFP is more structured at 5 °C, and support the general observation that AFPs become more rigid as the temperature is lowered but such phenomenon was not observed and analyzed at molecular level^47^.

In this research, we employed molecular dynamics simulation for spruce budworm antifreeze protein at low temperatures to gain insight into the changes in its structure and thermodynamics. Our computational result were then compared to experimental data for validation.

## Materials and Methods

### Materials

The 3D structure of spruce budworm antifreeze protein (sbAFP) was collected from Protein Data Bank (PDB code 1N4I)^47^. We used Visual Molecular Dynamics (VMD)^48^ to analyze and visualize the atomistic structure of sbAFP. The melting point of water models used in MD simulation did not echo fully the melting point of actual water^49^, e.g. the melting temperature of ice I(h) for several commonly used water models (SPC, SPC/E, TIP3P, TIP4P, TIP4P/Ew, and TIP5P) obtained from computer simulations at p = 1 bar. Since the melting temperature of ice I(h) for the TIP4P model was already known^50^, it was possible to use the Gibbs-Duhem methodology^51^ to evaluate the melting temperature of ice I(h) for other potential models of water. Previous studies found that the melting temperatures of ice I(h) for SPC, SPC/E, TIP3P, TIP4P, TIP4P/Ew, and TIP5P models were 190 K, 215 K, 146 K, 232 K, 245 K, and 274 K, respectively. In general, it is difficult to determine the freezing process of waters as well as the formation process of ice crystals at real temperatures. Therefore, the heterogeneous biphasic system composed of hydrophobic (cyclohexane) layer was generated to substitute for ice crystal structure. Acyclohexane structure (CHX) was built by GaussView 5.0 and its geometry was optimized by Gaussian 09^52^, then the topology was generated by PRODRG servers^53^,^54^ and modeled under the GROMOS96 43a1 force field^55^.

Finally, we used tools integrated in GROMACS 4.5.5 software^56^ to generate CHX layers and balance them through 10,000 ps simulation. The cyclohexane layers adequately demonstrated the properties of hydrophobic interactions. In this research, the cyclohexane layers were used as the representation for ice crystal to investigate the change of structure and thermodynamics of sbAFP under interaction of ice-water at low temperatures.

### Simulation Methods

**Figure 1:**
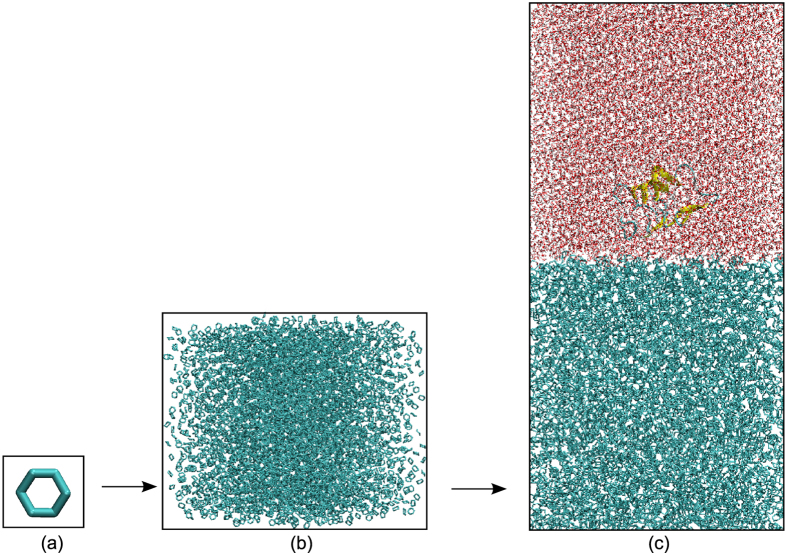
The image illustrates (**a**) a cyclohexane molecule (**b**) cyclohexane layer and (**c**) complex structure of a heterogeneous biphasic system composed of hydrophobic (cyclohexane) layer, hydrophilic (waters) layer and spruce budworm antifreeze protein (sbAFP).

The GROMACS 4.5.5 package^56^ was used to run MD simulations with the GROMOS96 43a1 force field^55^. The protein was placed in a triclinic box with the edges of 9, 9, and 18 nm which contains approximately 20044 water molecules of the SPC water model^57^ and 3728 cyclohexane molecules (Fig. 1). The center of the protein was placed at 4.5, 4.5, and 10.5 nm so that sbAFP was completely located in water layer. Na^+^ or Cl^−^ counter ions were added using tool in the GROMACS 4.5.5 package in order to neutralize the systems. The LINCS algorithm^58^ was used to constrain the length of all bonds. The vdW forces were calculated with a cutoff of 1.4 nm, and particle-mesh Ewald method^59^ was employed to treat the long-range electrostatic interactions. The non-bonded interaction pair-list was updated every 1 fs with the cutoff of 1.2 nm. Upon the completion of steepest descent minimization, position-restrained MD simulations were performed for 500 ps so that water molecules move into protein’s pockets. The whole system was then gradually heated from 0 to 300 K during 500 ps of the MD run. To ensure the complex reaches stability, the system was equilibrated for 1000 ps at 300 K in the canonical ensemble (NVT - amount of substance (N), volume (V) and temperature (T)) run using the Berendsen procedure^60^, and subsequently in 1500 ps isothermal-isobaric ensemble (NPT - amount of substance (N), volume (V) and temperature (T)) run at pressure of 1 bar using the Parrinello-Rahman pressure coupling^61^. The structures were generated as the configurations for our MD simulation with 8 temperatures including 268 K, 273 K, 278 K, 283 K, 288 K, 293 K, 298 K and 303 K. The final MD simulation allowed us to integrate the equations of motion with a time step of 1 fs and our simulation run for totally 10,000 ps in the leap frog algorithm^62^.

## Results and Discussion

In previous publications, the experimental methods (X-Ray diffraction, Neutron diffraction or solution NMR) and molecular dynamics simulations (atomistic or coarse grained models) are used to study the freezing process of water molecules surrounding AFPs at low temperatures^3^,^24^,^30^,^31^,^39^,^46^. The aqueous solution is observed to be frozen at extremely low temperature, formed the ice crystal network under the vaguely hexagonal shape due to the special bond angles within the water molecules as it forms a solid crystal lattice, and fully represented the characteristic of hydrophobic interaction^3^,^63^. The ice crystal network would be enlarged over the simulation time, and probably expand the influenced region of ice crystals in the whole simulation space. The collective motions, fluctuation, and relaxation dynamics of liquid water will be reduced and completely inhibited in order to form ice crystal (crystallization or recrystallization process). This is similar to the hydrophobic interaction between AFPs and the environment was increased when bulk water surrounding AFPs transferred from liquid form to ice crystal at low temperatures^3^,^30^,^31^ or AFPs transferred from liquid water region to CHX region. Therefore, the characteristics of hydrophobic and hydrophilic interactions at ice-binding and non-ice-binding sites of sbAFP in environment containing water and CHX molecules were used to investigate sbAFP’s activity at low temperatures.

**Figure 2:**
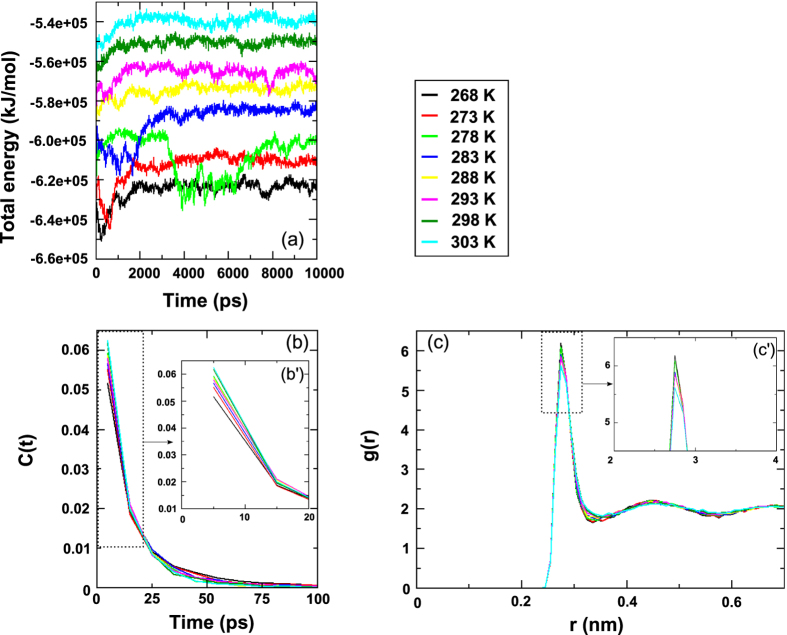
The image illustrates total energies of the studied complexes (**a**), hydrogen bond lifetime correlation C(t) for water molecules around sbAFP (**b**) and the radial distribution function of water-water in sbAFP complexes (**c**) at different temperatures. The values revealed the activity of sbAFP under temperature variations.

The variation in total energy of sbAFP was shown as function of simulation time to determine the activity of sbAFP on variation of freezing temperatures, and when subfreezing temperatures increased, the total energies also increased. As shown in Fig. 2a, the total energy of sbAFP systems reached stability in accordance with temperature setup as simulation time increased. In general, the total energy of those systems was found to reach stability after 1000 ps for 268 K, 273 K, 288 K, 293 K, 298 K and 303 K; 2000 ps for 283 K; and 8000 ps for 278 K. In detail, there are two energy status for the complexes at temperatures of 268 K, 273 K, 283 K, 288 K, 293 K, 298 K and 303 K; it is ranged from 0 ps to 2000 ps for unstable and in stable for remaining time of simulation processes. The total energy of the complex simulated at 278 K totally matched with four energy statuses including first status from 0 ps to 1000 ps for unstable, second status from 1000 ps to 3500 ps for stable, third status from 3500 ps to 7500 ps for unstable, and fourth status from 7500 ps to 10000 ps for stable. With many energy status of sbAFP structure at 278 K, the result indicated that sbAFP was more structured and supported the general observation that sbAFP became more rigid as the temperature was lowered. This agreed with experimental results in prior publication^47^. In summary, the results demonstrated the activities of water and CHX molecules surrounding sbAFP at subfreezing temperature in which the formation and expansion of hydrophobic interaction at low temperatures led to the decrease of thermodynamic properties of the complexes. The activity of sbAFP during simulation was demonstrated more detail in the hydrogen bond life (C(t)), the radial distribution function (g(r)) and the mean square displacement (MSD) parts.

In an effort to display the remarkable difference between the water dynamics in the vicinity of the ice-binding and the non-ice-binding sites of sbAFP, the hydrogen bond lifetimes (C(t)) between water molecules and the sbAFP, distributed over the surface of sbAFP, were measured with the longest lifetimes on average on the ice-binding site. As seen from Fig. 2b, the C(t) reached a value of 0.2 for hydrogen bonds between water and the atoms of the ice-binding site at 15 ps and was also phase transition point between two status of stable and unstable. Then C(t) is advanced to 0 for hydrogen bond between water and the atoms at the ice-binding site after 100 ps. In more details, Fig. 2b′ is also shown that the C(t) value at 278 K is fluctuated near 298 K and 303 K, meanwhile, the other values are increased accordingly with temperatures. The result, therefore confirms that sbAFP was more structured at this temperature. In general, the finding was a clear trend of retardation of the hydrogen bond dynamics, with a gradient to the ice-binding site.

The radial distribution function g(r) was used to describe the behavior of water molecules around sbAFP under subfreezing temperatures. The g(r) was measured to investigate the distance of water molecules that came into contact with sbAFP. At subfreezing temperatures, the g(r) exhibited long range correlation due to the reduction in water mobility. Specifically, Fig. 2c shows the radial distribution function of water-water around sbAFP at simulated temperatures and Fig. 2c′ is shown clearer about the g(r) values of water-water around sbAFP of only first peaks. The results show a tendency to increase peak height when temperature decreased, and the peak’s height are fluctuated around 6 for the first peak, 2.5 for the second peak and 2.2 for the third one. The fact that peak height decreased as temperature increased reflected the changes of sbAFP caused by its surrounding at lower temperatures, this means the decrease of hydrophilic interaction, led to the increase of hydrophobic interaction at lower temperatures, did alter structure and thermodynamics of sbAFP. Here, the g(r) of CHX did not display distinguishable differences in the liquid structure because it just expressed the structure and property of the ice network and did not significantly influence the configurations of CHX in the bulk liquid.

**Figure 3:**
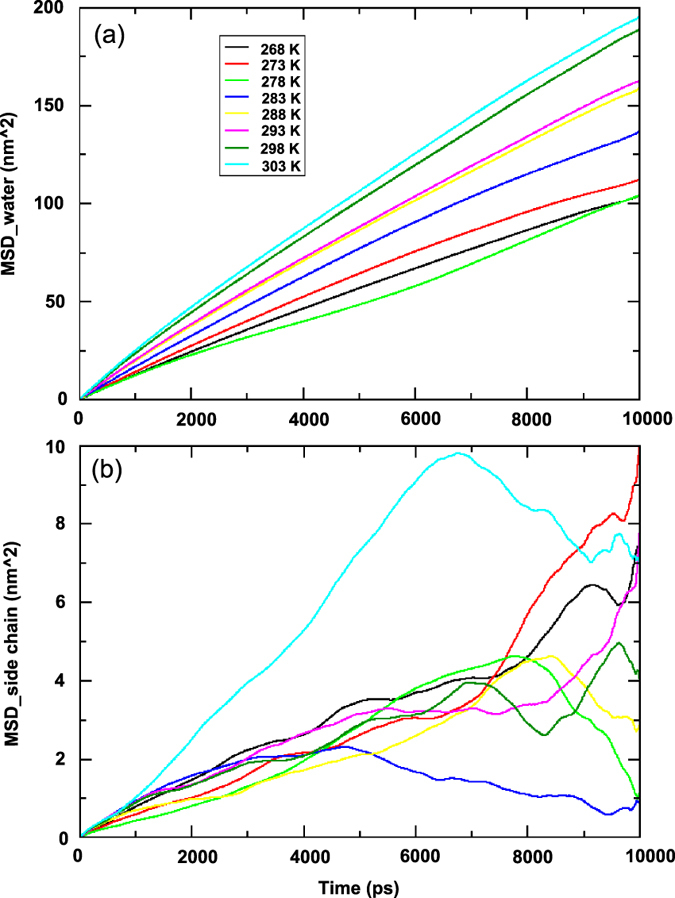
Mean square deviation analysis of ice-water molecules (**a**) and side chain atoms (**b**) gained by MD at eight simulation temperatures.

The mean square displacement (MSD) was used to determine fundamental thermodynamic quality and the temperature dependence of MSD to characterize the internal flexibility of water molecules as well as protein. The MSD values were increasing in accordance with their corresponding temperatures. The MSD plot depicts the thermal fluctuation of atomic displacement from the average position. These fluctuations are references to the center of mass, which reveals better convergence criteria. The differences in MSD calculated by the two approaches are due to the presence of large number of degrees of freedom for all atom analysis. MSD provides information on diffusion property of solutes, which tends to increase rapidly with increasing temperature. MSD plot represents the motion of sbAFP according to the potential antifreeze molecules. Figure 3a shows the mean square displacement result of water molecules surrounding sbAFP. Here, the MSD values were increased when the temperatures increased, except for the complex simulated at 278 K due to more structured property of sbAFP presented in prior parts. Particularly, the side chain diffusion pattern for sbAFP at simulation temperatures were more prominent as described by MSD of side chain in Fig. 3b. The data indicate that the water trapping times in local minima across the potential surface of ice crystal is higher at lower temperature but it is not reviewed for the case of complex at 278 K. So, it can be found that the diffusive behavior of the control sbAFP is optimum at 278 K for its side chain. This result reveals that sbAFP has a special adaptive mechanism at 278 K, which most of the other antifreeze proteins do not have.

**Figure 4:**
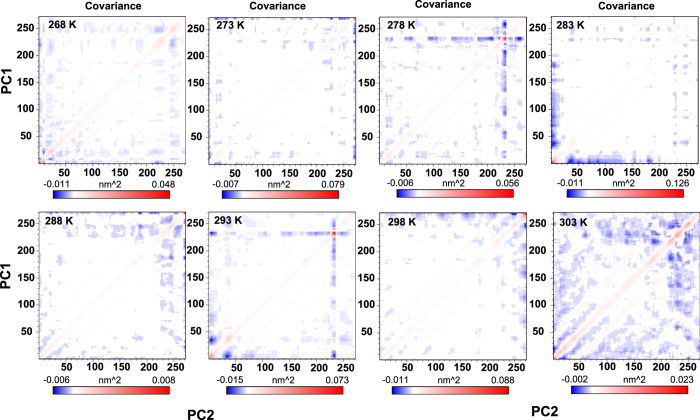
The principal component analysis (PCA) for protein conformation reveals unique behavior in specialized MD simulation.

Principal component analysis (PCA) for protein conformation was used to explain the maximum amount of variance with the fewest number of principal components, and determine the motions in contributing most of the overall thermodynamics of protein. In a complex including N atoms, there exist 3 N + 6 models of possible internal fluctuations (6 degrees of freedom required for the description of the external rotation and translations). The conformational space spanned by sbAFP simulation temperatures were conserved the correlated projection of the PC1 and PC2 (protein backbone atoms) and the axis scale was fixed for all scatter plot representations. As shown from Fig. 4, the PCA’s result at different temperatures from 268 K to 303 K shows that protein conformation attained a sufficient degree of atoms to constitute many different configurations. The PCA result is close to the realistic condition as sbAFP in the presence of ice reflects its physiological relevance. The significant changes of magnitude in the distribution of PCA according to the PC1 and PC2 axis were extended when temperature is increased. In detail, most of the sbAFP’s activities were inhibited by hydrophobic interaction (CHX region) at low temperatures lead to limit to formation process of sbAFP’s configurations (at temperatures which are lower and equal to 273 K). Therefore, when temperature was increased up to 303 K, it was more advantageous for sbAFP to be activated and to form many different configurations due to the effects of hydrophilic interactions (in water region). It was observed that sbAFP formed most of its configurations at 303 K and fewest configurations at 268 K.

**Figure 5:**
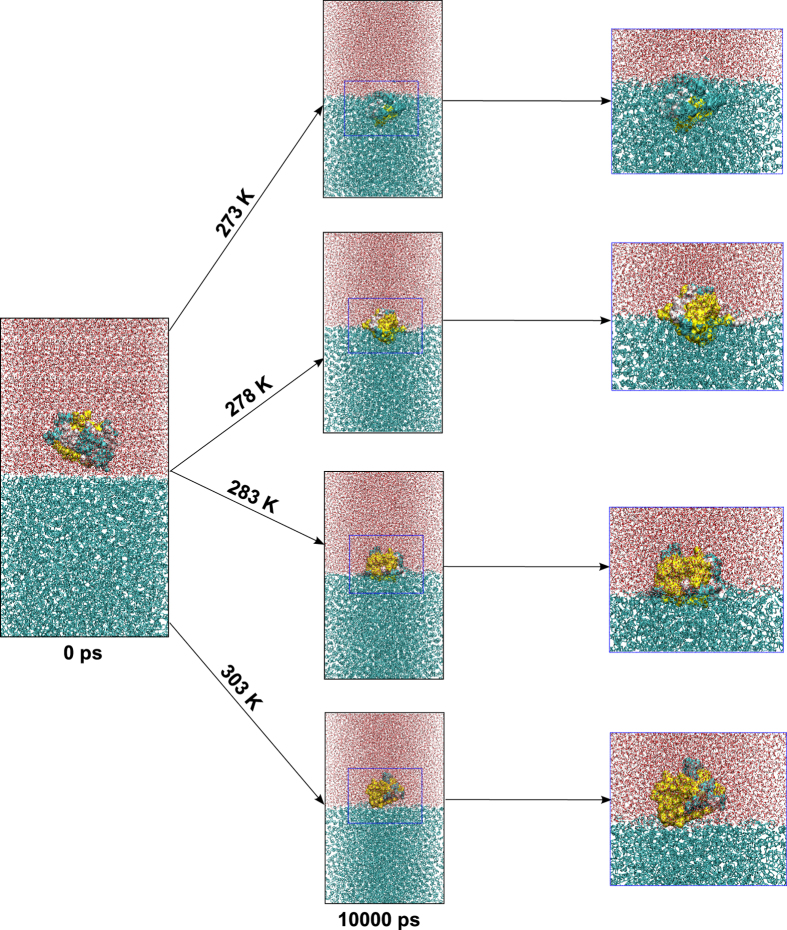
The snapshots show the changes of sbAFP in water-CHX environments at temperatures of 273** **K, 278** **K, 283** **K and 303** **K over simulation time.

The simulations provide a clue as to how the freezing transition happens at lower temperature for sbAFP. Figure 5 shows snapshots of sbAFPP and surrounding water-CHX molecules during the growth of hydrophobic interaction and sbAFP partially surrounded by the growth of hydrophobic interaction during the simulation. Here, the snapshots just show the complex structures at 0 ps and 10000 ps for four different temperatures of 273 K, 278 K, 283 K and 303 K, and the snapshots of sbAFP complexes included water molecules, CHX molecules, Na^+^ and Cl^−^ ions and protein in simulation complexes. At 0 ps, sbAFP is completely located in water environment, protein-water interaction played an important role in explaining by sbAFP’s activity, here, the hydrophilic interaction was key interaction. At 10000 ps, the snapshots show the difference between hydrophobic and hydrophilic interactions for the complexes at the temperatures of 273 K, 278 K, 283 K, and 303 K, the results indicated that: For the simulated complex at 273 K, sbAFP is located deep into CHX region and only a small part at non-ice-binding site of sbAFP was in contact with water region. This means that the hydrophobic interaction was the key factor in the interaction between sbAFP and surrounding environment or this also means sbAFP protein was covered by ice network, thus sbAFP’s activity was completely inhibited at this temperature. For the complex simulated at 278 K, almost a half of sbAFP was located in CHX region and the remaining part is located in water region, here, the hydrophobic and hydrophilic interactions could be equally contributed, which corresponded to the ice-liquid hybrid status that Hung Nguyen *et al*. previously reported^3^,^28^,^29^. For the complex simulated at 283 K and 303 K, sbAFP was covered by bulk water and only a small part at ice-binding site of sbAFP was in contact with CHX region. This means that the hydrophilic interaction was the main interaction which accounted for most of the sbAFP’s activity in the environment, as mentioned above, and sbAFP was not inhibited by hydrophobic interaction and functioned better at temperatures larger than 283 K. In general, the changes of hydrophobic and hydrophilic interactions at ice-binding and non-ice-binding sites of sbAFP depended on simulation temperatures, and could be specially adapted mechanisms of sbAFP structure to environment at subfreezing temperatures.

## Conclusions

Molecular dynamics simulation was applied to investigate the changes in structure, thermodynamic and adapted mechanisms of sbAFP under various subfreezing temperatures. We have obtained a number of interesting results as follows: (1) sbAFP has some special changes in structure and interaction with water and CHX regions at 278 K, as being transition temperature point of water molecules in sbAFP complex at low temperatures, which is more structured and support the general observation to become more rigid as the temperature is lowered, the result is strongly agreement with experimental results. (2) The change of hydrophobic and hydrophilic interactions at ice-binding and non-ice-binding sites of sbAFP depended on simulation temperatures, and could be a specially adapted mechanisms to cold environment of sbAFP structure at low temperatures. (3) Our result points out that sbAFP has the capability to inhibit the freezing process of water at low temperatures via hydrophobic and hydrophilic interactions and that sbAFP was also found to be energetically comparable and exhibited antifreeze property at subfreezing temperature of greater 283 K.

## Additional Information

**Accession codes:** The coordinates and structure factors have been deposited in the Protein Data Bank with accession code 1N4I.

**How to cite this article**: Nguyen, H. and Le, L. Investigation of changes in structure and thermodynamic of spruce budworm antifreeze protein under subfreezing temperature. *Sci. Rep.* **7**, 40032; doi: 10.1038/srep40032 (2017).

**Publisher's note:** Springer Nature remains neutral with regard to jurisdictional claims in published maps and institutional affiliations.
